# Protein and Proteome Atlas for Plants under Stresses: New Highlights and Ways for Integrated Omics in Post-Genomics Era

**DOI:** 10.3390/ijms20205222

**Published:** 2019-10-21

**Authors:** Xuchu Wang

**Affiliations:** Key Laboratory for Ecology of Tropical Islands, College of Life Sciences, Ministry of Education, Hainan Normal University, Haikou 571158, China; xchwang@hainnu.edu.cn; Tel.: +86-898-6589-1065

**Keywords:** integrated omics, plants under stress, post-genomics era, proteome atlas, quantitative proteomics

## Abstract

In the post-genomics era, integrative omics studies for biochemical, physiological, and molecular changes of plants in response to stress conditions play more crucial roles. Among them, atlas analysis of plants under different abiotic stresses, including salinity, drought, and toxic conditions, has become more important for uncovering the potential key genes and proteins in different plant tissues. High-quality genomic data and integrated analyses of transcriptomic, proteomic, metabolomics, and phenomic patterns provide a deeper understanding of how plants grow and survive under environmental stresses. This editorial mini-review aims to synthesize the 27 papers including two timely reviews that have contributed to this Special Issue, which focuses on concluding the recent progress in the Protein and Proteome Atlas in plants under different stresses. It covers various aspects of plant proteins ranging from agricultural proteomics, structure and function of proteins, novel techniques and approaches for gene and protein identification, protein quantification, proteomics for post-translational modifications (PTMs), and new insights into proteomics. The proteomics-based results in this issue will help the readers to gain novel insights for the understanding of complicated physiological processes in crops and other important plants in response to stressed conditions. Furthermore, these target genes and proteins that are important candidates for further functional validation in economic plants and crops can be studied.

With the annotation of genomes for human [1] and hundreds of plant species, plant biology study is dawning upon the post-genomics era [2]. Biochemical, physiological, and molecular studies have paved the way toward a comprehensive understanding of the complex biological processes for plants in response to stress conditions. Among them, abiotic stresses are the foremost limiting factors for plant survival and development [3]. Different from animals, plants cannot move away from the stressed conditions and they must cope with all kinds of adverse external pressures via their intrinsic biological mechanisms [3]. In this new post-genomics time, atlas analysis of plants under different abiotic stresses, including salinity, water logging, cold, drought, heat, UV radiation, heavy metals, anaerobic, and toxic conditions in the root zone, among others, has become more important for uncovering the potential key genes and proteins in different plant tissues [4]. High-quality genomic data and integrated analyses of transcriptomic, proteomic, metabolomics, and phenomic patterns (Figure 1) provide a deeper understanding of how plants grow and survive under environmental stresses [5]. 

We organized sixteen researchers in the omics study area to edit this Special Issue titled “Plant Protein and Proteome Atlas: Integrated Omics Analyses of Plants under Abiotic Stresses”, and this Special Issue book is edited based on these published papers. Therefore, this special book is focused on concluding the recent progress in the Protein and Proteome Atlas in plants under different stresses. Integrated omics analysis covers various aspects of plant protein ranging from agricultural proteomics, structure and function of proteins, novel techniques and approaches for gene and protein identification, protein quantification, proteomics for post-translational modifications (PTMs), and new insights into proteomics. Proteome atlas aims to compare the quantified relative abundances of the genome-wide genes and proteins across different plant tissues or subcellular compartments. Large-scale analyses of post-translational modifications in proteins, such as phosphoproteomics, glycoproteomics, and ubiquiproteomics, have become more imperative to define and interpret the plant–environment relationships in terms of protection against abiotic stresses in multi-layers [5]. It helps to gain novel insights into the identification of target genes and proteins, which may decipher the complex relationship between genes, proteins, metabolites, and their biological functions. At the same time, combining the big-data-based multi-omics approaches and traditional molecular biology technologies gains deeper insights into the stress-mitigating mechanisms in plants for translation into higher productivity [5].

In this issue, we included crop plants as well as model plant species for fundamental research of stress physiology and biochemistry. The topics focus on integrative analyses of quantitative protein change in plants under abiotic stresses; transcriptomic, proteomic, metabolomics, and phenomic analyses of plant species and tissues under abiotic stresses; plant proteome atlas of different tissues and cell compartments; post-translational modifications in plant proteins upon stressed conditions; bioinformatics and computational tools for analyzing big data via various omics approaches; genetic and phenomic studies of plant species in different environments; and identification and functional validation of key genes and proteins obtained from integrative omics approaches in response to stresses in plants. 

Finally, a total of 27 papers including two timely reviews were divided into five parts in this special book. Part 1 was edited by Dr. Xuchu Wang from Hainan Normal University. This part contains six papers describing the comparative proteomics of different plants under different conditions. Proteomics analyses were performed on six plant species, including spinach [6], *Brassica rapa* [7], wheat [8], diatom [9], lily [10], and a rubber grass *Taraxacum Kok-saghyz* [11], in order to gain deeper insights into the stress-mitigating mechanisms in plants under different conditions. Li et al. (2019) performed a comparative proteomics analysis of the leaves under both low-temperature and high-temperature stressed conditions in heat-sensitive spinach and identified 257 heat-responsive proteins [6]. Their proteomics data revealed that both the photosynthesis process and reactive oxygen species scavenging pathways are inhibited in response to high temperature stress. A similar proteomics study performed by Yuan and colleagues resulted in 1022 differentially expressed proteins in Wucai under temperature stressed conditions, and most of these proteins were identified to be associated with redox homeostasis, photosynthesis, carbohydrate metabolism, and heat-shock response [7]. In wheat plants, pollen, as a highly specialized organ, develops in the anther. Comparative cytological and proteomic analyses were conducted by Wang et al. (2019) to better understand the mechanism on the chemical hybridizing agent induced pollen abortion in wheat, and determined 60 significant different proteins [8]. Furthermore, Thangaraj et al. (2019) performed an iTRAQ-based proteomics analysis to explore the distinct cellular responses associated with oxidative stress in the diatom *Skeletonema dohrnii*, and determined 594 differentially expressed proteins from 1768 proteins. Their proteomics data also revealed that ATP-limited diatoms are unable to rely on photosynthesis, owing to break down of carbon metabolism to compensate for photosynthetic carbon fixation losses [9]. Comparative proteomics were used to investigate the relationship between hydrogen gas and NO, and identified 50 differentially accumulated proteins during postharvest freshness in the Cut Lilies leaves [10]. Xie and coworkers [11] provided a proteomic landscape of the mature roots of a rubber-producing grass *Taraxacum Kok-saghyz* and identified 371 protein species from the mature roots by combining 2-DE and MS. Meanwhile, 3545 individual proteins were determined by a large-scale shotgun proteomics analysis of the enlargement roots, and fifty-eight natural rubber biosynthesis-related proteins were identified; these proteins were involved in both mevalonate acid and methylerythritol phosphate pathways [11]. This is the first high-resolution draft proteome map of the mature roots of rubber grass.

Furthermore, proteomics analyses of different plants under both salinity and drought stresses were conducted to find the osmotic response proteins [12,13,14,15,16,17]. Drought and salinity are two serious kinds of osmotic stresses that inhibit plant growth and crop yields. Recent comparative proteomics analyses have provided more information for understanding the drought- and salinity-responsive mechanisms in certain plant species. In this part, we included six papers and focused on proteomics analyses of plants under osmotic stress. Drought, as an important abiotic stress, can seriously limits crop yields. Wang and colleagues adopted iTRAQ and LC/MS approaches to determine the different proteins in wheat leaves after exposure to PEG, and finally identified 176 differentially expressed proteins [12]. In another drought-tolerant wheat variety, 335 drought-responsive proteins were found to mainly take part in photosynthesis, carbon fixation, and glyoxylate and dicarboxylate metabolism [13]. In addition, a comparative analysis of filling-kernel proteomes from two maize inbred lines with different drought-tolerant ability identified 5175 drought-responsive proteins [14]. Proteomics study implied that wood vinegar treatment can enhance the drought tolerance of wheat root through promoting stress response, carbohydrate metabolism, protein metabolism, and secondary metabolism [15]. Two proteomics studies have been performed to find the stress-responsive proteins in the leaves of *E. angustifolia* seedlings [16] and *D. salina* during early response to salt stress [17], which implied that the symbiosis of halophytes and arbuscular mycorrhizal fungi has potential as an application for the improvement of saline-alkali soils. An iTRAQ-based proteomics analysis of *D. salina* revealed that photosynthesis and ATP synthesis are crucial for the modulation of early salinity-responsive pathways [17]. Improving osmotic tolerance of crops to salinity and drought stress has not yet been realized by molecular engineering, and proteomics of plants under salinity and drought stress present an elaborate understanding of stress-responsive proteins and metabolic pathways. These osmotic-response proteins and genes are important candidates for further functional validation.

Plant propagation and development are two important aspects that could largely determine the life cycle and economic values of different crops. The special issue studied different plants species, including an energy plant *Jatropha curcas* [18], a biofuel tree Pongamia [19], and three economic crops [20,21,22]. Liu et al. used combined analyses of the phosphoproteomics, physiological characteristics, and ultrastructure studies to identify the responses of *J. curcas* seedlings under chilling, and revealed significantly changed phosphoproteins under chilling stress [18]. A comparative study between the salt resistance and sensitive cultivars with very close genetic background will undoubtedly help to explore the key regulator. On the basis of this idea, Zhao et al. conducted a phosphorproteomic analysis between two maize inbred lines showing different resistance to salt stress, and found that the enhancement of potassium and sodium transportation, carbon, and redox-related metabolism could increase the salt resistance in maize [19]. In this issue, Yu et al. applied a newly developed Phos-tagTM technology to identify 21 phosphorylated peptides of AGPase [20]. 

Cotton is the most important natural fiber resource in the world, which makes it an important crop. Studies have been widely conducted focusing on many aspects of this crop. Although genetic modified cotton cultivars have been generated and widely used in its production, transformation of cotton could only be succeeded in very limited germplasm with low efficiency. It is very important to identify the key regulators that determine the fate of somatic embryos. Comparative proteomics were successfully applied by Guo et al. to determine 6730 proteins in the embryogenic calli of cotton [21]. Additionally, Lin and colleagues reviewed the latest advancement studies of lotus and provided more omics information on this plant [22].

Integrative omics tools, including genomics, transcriptomics, proteomics, and metabolomics, are very powerful to study the molecular basis of biological activity between biomolecules (DNA, RNA, proteins, and metabolites). Integrative omics methods have been widely used to study biomolecules for their interactions; biosynthesis; and the regulation of these interactions in the various systems of plants, plant development, and their interaction with various environments. Li and coworkers combined physiological and proteomic methods to study the changes in alligator weed stems under low potassium stress, and provided valuable information on the adaptive mechanisms in alligator [23]. Using combined metabolomic and transcriptomic analyses, Guo et al. revealed that dynamic regulation of purine metabolism and flavonoid synthesis in transdifferentiation during somatic embryogenesis is crucial for cotton regeneration [24]. A total of 581 metabolites were present in the embryogenic calli, and metabolites related to purine metabolism were significantly enriched. These omics data provide a valuable foundation for a deeper understanding of the regulatory mechanisms underlying cell totipotency at the molecular and biochemical levels.

Furthermore, many stress responsive genes were identified by transcriptomics analyses [25,26,27]. Dong and coworkers discovered that the leaf sheath transcriptome has dynamic perturbation, and the processes and genes involved in sheath maturation are important for organ specialization [25]. Yang and coworkers discovered differentially expressed mRNAs and potential pathways involved in heat stress in radish leaves. They detected 1802 differentially expressed mRNAs and 169 differentially expressed lncRNAs, as well as three differentially expressed circRNAs, through strand-specific RNA sequencing technology [26]. Jin et al. unraveled 1327 microRNA-mediated genes in the regulation of Pongamia seeds by high-throughput small RNA profiling and identified 236 conserved miRNAs and 143 novel miRNAs within the families by deep sequencing of Pongamia seeds [27]. These results provide valuable miRNA candidates for further functional characterization and breeding practice in Pongamia and other oilseed plants.

More stress responsive genes and proteins in different plants have been identified, and their functions are studied in the following part of this Special Issue. With the development of sequencing technology and molecular biology, studies on plant growth and development, environmental adaptation, and other mechanisms will no longer be restricted to physiological phenomenon, but will be more popular to use the omics-based methods to identify the stress responsive genes and proteins in different plants to decipher the intrinsic regulating mechanisms from the transcriptome and proteome level. Tang and colleagues explored a proteomics analysis of the regulatory mechanism of nitrogen fertilization in cereal crops, and determined 511 differentially expressed proteins among the identified 6093 proteins in the two rice cultivars [28]. Micro-exons, a set of small exons with lengths no more than 51 nucleotides, have been widely studied in recent years. Song and coworkers investigated the potential functions of micro-exons in the whole genome of two *indica* rice varieties [29].

Polyamines play an important role in plant growth and development, as well as response to abiotic stresses. Ji and coworkers applied proteomics of Sugar Beet M14 under salt stress, and found that S-adenosylmethionine decarboxylase can enhance salt tolerance via mediating the biosynthesis of spermidine and spermin [30]. Myo-inositol-1-phosphate synthase plays important roles in plant growth and development, stress responses, and cellular signal transduction. Ma and coworkers proved that Myo-inositol-1-phosphate synthase can rescue the abnormal phenotype and promote the root cell elongation [31]. Inositol signaling is believed to play a crucial role in various aspects of plant growth and adaptation. A mini-review on the function of inositol phosphatases in plant tolerance to abiotic stress was included. In this timely review, the authors concluded the functions of more than 30 members of inositol phosphatases in plants, and revised some current knowledge in relation to their substrates and function in response to abiotic stress [32].

We believe that the proteomics-based results in this issue will help the readers to gain novel insights for the understanding of complicated physiological processes in crops and other important plants in response to stressed conditions. The identification of target genes and proteins will decipher the complex relationship between genes, proteins, metabolites, and their biological functions in plants, and these genes and proteins are important candidates for further functional validation and may provide valuable information for further study in molecular breeding in economic plants and crops.

## Figures and Tables

**Figure 1:**
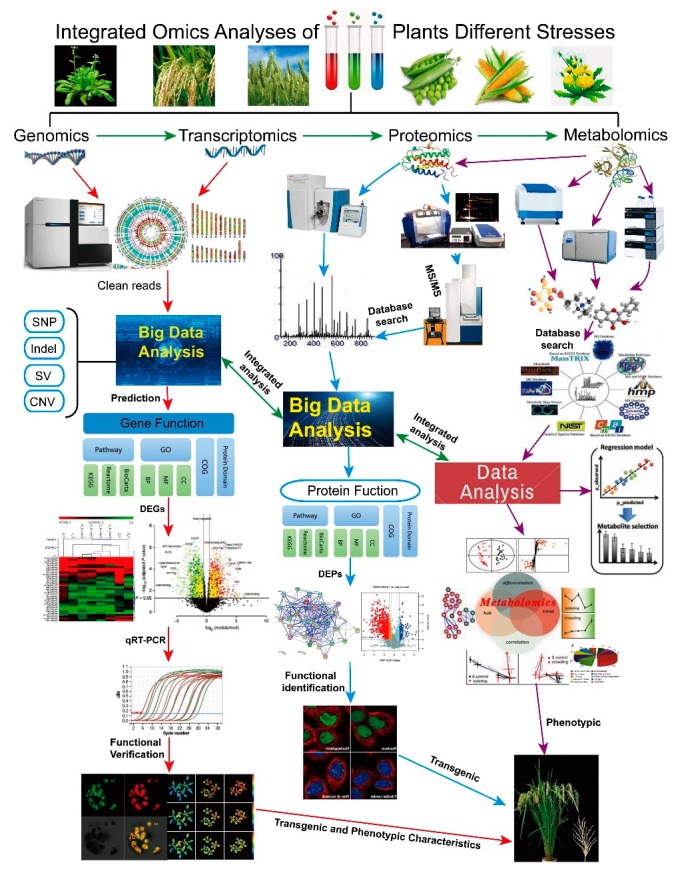
Integrated omics analyses of plants under different stresses. On the basis of the high-quality genomic data, integrated analyses using transcriptomic, proteomic, metabolomics, and phenomic methods have recently been performed in different plant species under different biotic and abiotic stressed conditions to determine their stress responsive genes and proteins, after which functional analyses of these target genes and proteins are conducted by molecular and biochemical methods. These integrated data have provided a deeper understanding of how plants grow and survive under different environments.
